# Mississippi Valley Dental Association

**Published:** 1871-04

**Authors:** 


### Editorial.

### MISSISSIPPI VALLEY DENTAL ASSOCIATION.

The last meeting of this old and established association was well attended, both as regards the enrollment and the quality of the members present. A full report of the discussions is published in the present number of the Register, and they will speak for themselves.

The discussions were not as full as could be desired upon any of the subjects presented, and it is just possible that little or no thoughts was bestowed in preparing them for associative judgment. Herein lies one of the elements of futility, and its permitted existence is all the more deplorable and reprehensible when every member must feel that great benefit would accrue were a more systematic and united effort made to flood every subject for discussion, with the fullest light of intelligence and experience. The central idea of association is to be found, we opine, in this high light influx. The association to the professional man, is what the exchange is to the merchant, perhaps more, and just in proportion as the individual gives will he receive.

Let loose a thinker upon a body of men, and behold the result. Some are seduced into throwing their thoughts into the common stock, others are provoked to do the same, while all are stirred and aroused until all the intellectual bristles stand on end. A meeting of professional men should bristle with thought always, and it will fall short of purpose and usefulness when its members sink into mere routine and indifferency.

The meeting in question was noteworthy for elevated tone and the entire absence of personalties. A spirit of enquiry characterized the sessions, and the members separated, feeling that the time was well spent, and that no matter how far experience may take the individual, he will still obtain glimpses beyond, through the experience of others. T.

